# Expression profiles of urbilaterian genes uniquely shared between honey bee and vertebrates

**DOI:** 10.1186/1471-2164-10-17

**Published:** 2009-01-12

**Authors:** Toshiaki Matsui, Toshiyuki Yamamoto, Stefan Wyder, Evgeny M Zdobnov, Tatsuhiko Kadowaki

**Affiliations:** 1 Department of Applied Biological Sciences, School of Agricultural Sciences, Nagoya University, Chikusa, Nagoya 464-8601, Japan; 2 Department of Genetic Medicine and Development, University of Geneva Medical School, 1 rue Michel-Servet, 1211 Geneva, Switzerland; 3 Swiss Institute of Bioinformatics, 1 rue Michel-Servet, 1211 Geneva, Switzerland; 4 Imperial College London, South Kensington Campus, SW7 2AZ, London, UK; 5 Graduate School of Bioagricultural Sciences, Nagoya University, Chikusa, Nagoya 464-8601, Japan

## Abstract

**Background:**

Large-scale comparison of metazoan genomes has revealed that a significant fraction of genes of the last common ancestor of Bilateria (Urbilateria) is lost in each animal lineage. This event could be one of the underlying mechanisms involved in generating metazoan diversity. However, the present functions of these ancient genes have not been addressed extensively. To understand the functions and evolutionary mechanisms of such ancient Urbilaterian genes, we carried out comprehensive expression profile analysis of genes shared between vertebrates and honey bees but not with the other sequenced ecdysozoan genomes (honey bee-vertebrate specific, HVS genes) as a model.

**Results:**

We identified 30 honey bee and 55 mouse HVS genes. Many HVS genes exhibited tissue-selective expression patterns; intriguingly, the expression of 60% of honey bee HVS genes was found to be brain enriched, and 24% of mouse HVS genes were highly expressed in either or both the brain and testis. Moreover, a minimum of 38% of mouse HVS genes demonstrated neuron-enriched expression patterns, and 62% of them exhibited expression in selective brain areas, particularly the forebrain and cerebellum. Furthermore, gene ontology (GO) analysis of HVS genes predicted that 35% of genes are associated with DNA transcription and RNA processing.

**Conclusion:**

These results suggest that HVS genes include genes that are biased towards expression in the brain and gonads. They also demonstrate that at least some of Urbilaterian genes retained in the specific animal lineage may be selectively maintained to support the species-specific phenotypes.

## Background

Cross-species comparison of genome sequences and expressed sequence tag (EST) data sets have given us enormous insight into the evolution of metazoan genomes. In particular, the recent completion of a number of ecdysozoan and deuterostome genome projects as well as the collection of massive EST data on lophotrochozoan and cnidarians enable us to identify orthologous groups of genes and gene losses in these 4 major metazoan clades. Research on *Acropora millepora *(anthozoa, cnidaria) supports the view that a significant proportion of genes present only in the vertebrates and absent in model invertebrates is not a vertebrate-specific evolutionary change. Instead, these genes appear to have been lost in the specific metazoan lineages that branched from common ancestors during evolution [1]. The same conclusion is reached by considering the fact that sea anemone *Nematostella vectensis *contains many Wnt subfamilies that were lost from *Drosophila *and *Caenorhabditis *[2-4]. The cross-species comparison of *Aplysia californica *EST data sets with other metazoan genomes and with *Platynereis dumerilii *demonstrate that gene loss and sequence divergence are most extensive in model ecdysozoans (fruit fly and nematode), and lophotrochozoans are less derived from the complex ancestral genome of Urbilateria [5,6]. Urbilateria is a common ancestor of Bilateria, and 3 major clades (ecdysozoa, lophotrochozoa, and deuterostome) subsequently branched off from it [7].

Honey bees belong to the Hymenoptera, one of the 4 large holometabolous insect orders. They are highly social insects and have been used as a model system to study complex animal behavior and cognitive ability comparable to those of some vertebrates [8-10]. Furthermore, caste differentiation (differentiation of fertile queen and sterile worker, polyphenism) and sex determination via haplodiploidy (haploids and diploids develop into males and females, respectively) are specific to some hymenopterans such as honey bees, among the holometabolous insects. The completion of genome sequencing of several species in the 4 major holometabolous insect orders (Diptera, Lepidoptera, Hymenoptera, and Coleoptera), namely fruit flies (12 *Drosophila *species) [11], mosquitoes (*Anopheles gambiae *[12] and *Aedes aegypti *[13]), silk moth (*Bombyx mori) *[14], and honey bee (*Apis mellifera) *[15] and red flour beetle (*Tribolium castaneum) *[16] promises to provide new insights into how the genomes of holometabolous insects evolved in comparison to those of other metazoans. A comparison of the above insect genomes has revealed that a number of genes are lost in specific insects, but they are present in the other insects and vertebrates. These are the ancient genes of Urbilateria, which were retained in some species but lost from others. The loss and retention of these ancient genes in specific lineages are implicated in generating metazoan diversity; however, the causal relationships acting therein have not been studied extensively. We, therefore, identified genes shared between vertebrates and honey bees but absent in the other sequenced insect and nematode genomes, and we characterized their expression profiles in various honey bee and mouse tissues as well as the mouse brain to gain insight into their present functions in honey bees and mice. Furthermore, we expected that this study would give us clues to ascertain whether these genes have been selectively or randomly retained in the specific species during evolution. Our results demonstrate that the ancient Urbilaterian genes uniquely shared between honey bee and vertebrates are biased towards expression and functioning in the brain (often in specific regions) and testis. Their possible functions and evolutionary mechanisms will be discussed further.

## Results

### Identification of genes specifically conserved between vertebrates and honey bee but lost from the other sequenced ecdysozoan genomes

We identified candidate genes shared between at least 1 vertebrate (among *Homo sapiens*, *Mus musculus*, *Monodelphis domestica*, *Gallus gallus*, and *Tetraodon nigroviridis*) and the honey bee but not with 3 dipterans (*D. melanogaster*, *A. gambiae*, and *A. aegypti*) and a coleopteran (*T. castaneum*) species by automatic large-scale sequence analysis, as described in Methods [17]. We, then, performed a TBLASTN analysis of these candidate genes with the genomic and gene model sequences of the above insects as well as those of *B. mori *(Lepidoptera) and *C. elegans*. We discarded the ones that showed significant similarity with an E value < 1E-03. This screening resulted in the identification of 30 honey bee genes shared with vertebrates, but not with other sequenced insect and nematode genomes. We named them HVS (honey bee-vertebrate specific) genes, and they are listed together with the mouse homologs in Table 1. Twenty-one honey bee HVS genes encode proteins with known domains and/or functions. These include, for example, DNA methyltransferase Dnmt3 (GB14232), which has been reported previously [18]; homeodomain only protein (GB16549), which is highly expressed in the mouse brain [19] and heart [20,21]; Tuba (GB13871), which is a Cdc42-specific guanine nucleotide exchange factor to regulate actin polymerization [22]; and prenylcysteine oxidase 1 (prenylcysteine lyase, GB15533), a lysosomal enzyme degrading prenylcysteine [23].

**Table 1:** List of honey bee and mouse HVS genes

*Apis* GLEAN model	Mouse Accession Number	E-value	Name/Comments		Tissues highly expressed	
Proteins with known domains					Honey bee	Mouse

GB13403	NP_084053.2	2.00E-34	Rhbdd1	rhomboid domain containing 1	Ubiquitous	Ubiquitous
GB15999	NP_001001184.1	2.00E-60	Ccdc111	coiled-coil domain containing 111	Brain	Selective
GB16203	NP_083670.1	2.00E-12	Snx24	sorting nexing 24	Brain/Abdomen	Spleen
	NP_001020783.1	6.00E-12	Snx22	sorting nexing 22		Selective
GB17738	NP_598396.1	2.00E-53	Rtkn	rhotekin	Brain/Thorax	Selective
	XP_925652.1	3.00E-50	LOC631847	similar to pleckstrin homology domain containing, family K member 1		Lung
GB13871	NP_082305.1	8.00E-53	Tuba	dynamin binding protein	**Brain**	Selective
	NP_808496.1	6.00E-47	4933429F08Rik	hypothetical protein LOC328967		**Brain/Testis**
**GB14232**	**NP_001003963.1**	**4.00E-82**	**Dnmt3b**	**DNA methyltransferase 3B**	**Brain/Abdomen**	**Brain/Testis**
	**NP_031898.1**	**8.00E-74**	**Dnmt3a**	**DNA methyltransferase 3A**		Ubiquitous
GB15274	NP_033873.3	1.00E-09	Bcl2l1	Bcl2-like 1	Brain/Abdomen	Selective
	NP_803129	1.00E-07	Bcl2	B-cell leukemia/lymphoma 2		Selective
GB15533	NP_080099.1	3.00E-16	Pcyox1	prenylcysteine oxidase 1	**Brain**	**Brain**
	NP_766420.1	6.00E-16	Pcyox1l	prenylcysteine oxidase 1 like		Selective
**GB16549**	**NP_783199.1**	**9.00E-08**	**Hop**	**homeodomain only protein**	Ubiquitous	Selective
GB16908	NP_067303.1	4.00E-20	Bin3	bridging integrator 3	Brain	Selective
GB17445	NP_035789.1	5.00E-07	Tnfrsf4	tumor necrosis factor receptor superfamily, member 4	Brain/Abdomen	Selective
	NP_849262.1	1.00E-06	Tnfrsf14	tumor necrosis factor receptor superfamily member 14		Selective
GB14717	NP_001028486.1	1.00E-37	C530028I08Rik	hypothetical protein LOC232933	Brain	Testis
**GB15370**	**NP_080142.1**	**4.00E-41**	**Cdca7**	**cell division cycle associated 7**	**Brain/Thorax**	**Brain/Testis**
	**NP_666152.1**	**2,00E-40**	**Cdca7l**	**cell division cycle associated 7 like**		Selective
**GB14468**	**NP_067360.1**	**3.00E-50**	**Rad18**	**RAD18 homolog**	Brain	Testis
GB10755	NP_659185.1	4.00E-10	Tmem45b	transmembrane protein 45b	Brain	Selective
	NP_062605.2	6.00E-03	Tmem45a	transmembrane protein 45a		Selective
GB18761	NP_038957.1	2.00E-13	Siva1	Cd27 binding protein (Hindu God of destruction)	Brain	Ubiquitous
	XP_486223	6.00E-07	LOC434405	similar to Apoptosis regulatory protein Siva		Ubiquitous
GB10273	NP_082325.1	1.00E-11	Fbxo22	F-box protein 22	Brain	Ubiquitous
GB18346	XP_138107	9.00E-47	LOC217738	similar to thrombospondin, type I domain containing 3	**Brain**	**Brain/Testis**
	XP_996152	1.00E-45	5430433G21Rik	similar to isthmin 1		Lung
GB18120	NP_084460.1	5.00E-23	Armc9	armadillo repeat containing 9	**Brain**	**Brain/Testis**
GB18050	NP_083116.1	4.00E-09	Armc1	armadillo repeat-containing protein	Brain	Ubiquitous
GB19733	NP_056637.1	1.00E-50	Fbxl3	F-box and leucine-rich repeat protein 3	Brain	Ubiquitous
	NP_848789.2	1.00E-46	Fbxl21	F-box and leucine-rich repeat protein 21		Selective

Proteins without known domains						

GB18937	NP_080777.1	3.00E-06	1300010M03Rik	hypothetical protein LOC67998	Ubiquitous	Ubiquitous
	NP_001030023.1	8.00E-04	1810015C04Rik	hypothetical protein LOC66270		Selective
GB19146	NP_950182.1	2.00E-15	9230110C19Rik	hypothetical protein LOC234912	Brain	Selective
GB11010	NP_780695.1	1.00E-08	6430571L13Rik	hypothetical protein LOC235599	Brain	Selective
GB17260	XP_989436.1	1.00E-04	1700012P22Rik	hypothetical protein LOC69364	**Brain**	**Brain/Testis**
**GB16350**	**NP_081465.1**	**2.00E-11**	**Gemin7**	**gem (nuclear organelle) associated protein 7**	Ubiquitous	Ubiquitous
GB14271	NP_081498.1	1.00E-07	2010001M09Rik	hypothetical protein LOC69816	Brain	Selective
GB17835	NP_082206.1	1.00E-13	2610016C23Rik	DUF729 domain containing 1	**Brain**	**Brain/Testis**
	NP_080458.1	3.00E-09	Mtfr1	chondrocyte protein with a poly-proline region		Ubiquitous
**GB18344**	**NP_001028397.2**	**2.00E-08**	**Pnrc1**	**proline-rich nuclear receptor coactivator 1**	Ubiquitous	Brain
	**NP_080659.1**	**4.00E-07**	**Pnrc2**	**proline-rich nuclear receptor coactivator 2**		Ubiquitous
**GB15864**	**NP_899139.2**	**2.00E-05**	**Snapc5**	**small nuclear RNA activating complex, polypeptide 5**	Brain/Thorax	Selective

The genes categorized as transcription and modification of DNA as well as RNA processing by GO system are indicated with bold letters. The expression domains of honey bee and mouse HVS genes are listed in the right column. The genes ubiquitously and selectively expressed in the tissues are indicated as Ubiquitous and Selective, respectively. The genes highly expressed in the brains of both honey bee and mouse are shown with bold letters. The sequences of honey bee GLEAN models are available in BCM database  and BeeBase .

### Distribution patterns of HVS genes in sequenced deuterostome genomes

We analyzed the copy numbers of HVS genes among the sequenced deuterostome (*Strongylocentrotus purpuratus*, *Ciona intestinalis*, *T. nigroviridis*, *Xenopus tropicalis*, *G. gallus*, *M. musculus*, and *H. sapiens*) genomes, and the results are shown in Table 2. Deuterostomes appear to retain most of the HVS genes, in contrast to ecdysozoans. Although each HVS gene is present as a single copy in the honey bee genome, multiple-copy HVS genes are often present in the deuterostome genomes. All of the HVS genes, except Dnmt3; tumor necrosis factor (TNF) receptor superfamily; Tmem45; and F-box and leucine-rich repeat protein genes, are present as single copies in both sea urchin and ascidian genomes. Thus, the duplication of HVS genes is quite specific to vertebrate lineages. The vertebrates have 4–5 copies of Bcl2, one of the key regulators of apoptosis [24], and 4–9 copies of TNF receptor superfamily genes.

**Table 2:** Copy number of HVS genes in the deuterostome genomes

*A. mellifera*	*S. purpuratus*	*C. intestinalis*	*T. nigroviridis*	*X. tropicalis*	*G. gallus*	*M. musculus*	*H. sapience*
Genes encoding proteins with known domains							

GB13403	1	1	1	1	1	1	1
GB15999	1	1	1	1	1	1	1
GB16203	1	0	2	2	2	2	2
GB17738	1	1	2	2	1	2	2
GB13871	1	1	2	2	2	2	2
GB14232	1	2	3	2	2	2	2
GB15274	1	1	4	4	5	5	5
GB15533	1	1	2	2	2	2	2
GB16549	1	1	1	1	1	1	1
GB16908	1	1	1	1	1	1	1
GB17445	3	1	5	4	5	8	9
GB14717	1	1	1	1	1	1	1
GB15370	1	1	3	3	3	2	2
GB14468	1	1	1	1	1	1	1
GB10755	2	1	2	2	2	2	2
GB18761	1	1	1	1	1	2	2
GB10273	1	0	1	1	1	1	1
GB18346	1	1	2	2	2	2	2
GB18120	1	1	1	1	1	1	1
GB18050	1	1	3	1	1	1	1
GB19733	2	2	3	2	2	2	2

Genes encoding proteins without known domains							

GB18937	1	1	3	3	2	3	3
GB19146	1	1	1	1	1	1	1
GB11010	0	0	1	0	1	1	1
GB17260	1	1	0	1	1	1	1
GB16350	1	0	1	1	0	1	1
GB14271	0	1	0	0	0	1	1
GB17835	1	1	2	2	2	2	2
GB18344	0	1	2	1	2	2	2
GB15864	0	0	0	1	1	1	1

### Analysis of HVS genes by GO terms

We predicted HVS gene functions by assigning Gene Ontology (GO) terms (Gene-Ontology database, ) and found that 7 out of 20 gene products were proposed to be involved in transcription and modification of DNA as well as RNA processing in the nucleus. These include Dnmt3a/3b (involved in epigenetic control, [25]), Hop (transcriptional repressor, [20,21]), Rad18 (involved in DNA repair, [26]), Cdca7/Cdca7like, Pnrc1/2, Snapc5, and Gemin7. Gemin7 appears to be involved in RNA processing. The others are associated with the intracellular signaling cascade (for example, Rhotekin), regulation of apoptosis (Bcl2), actin filament organization (Bin3), receptor activity (TNF receptor super family), and ubiquitin cycle (Fbxo22). In *Drosophila*, proteins associated with the regulation of gene expression represent only 10% of the total proteins [27]. Considering that the total number of honey bee HVS genes is 30, it appears that HVS genes are biased to contain genes associated with these categories of cellular functions (Student *t*-test, *p *< 0.05).

### Expression profiles of HVS genes in honey bee and mouse tissues

**Figure 1:**
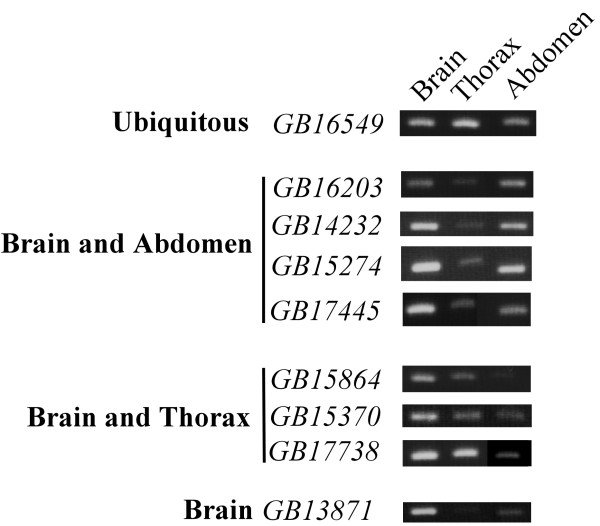
**Representative expression profile of HVS genes in honey bee tissues**. HVS mRNA levels in the honey bee brain, thorax, and abdomen were analyzed by semiquantitative RT-PCR. *GB16549 *and other (total 5 genes) mRNAs are ubiquitously expressed in the above tissues (see also Additional file 2). *GB16203*, *GB14232*, *GB15274*, and *GB17445 *mRNAs are expressed in the brain and abdomen but are less in the thorax. *GB15864*, *GB15370*, and *GB17738 *mRNAs are primarily detected in the brain and thorax. Expression of honey bee HVS genes, including *GB13871 *and others (total 18 genes) mRNAs was high in the brain (see also Additional file 2).

To confirm mRNA expression and to determine the functional implications of HVS genes, we carried out semiquantitative RT-PCR analysis for each HVS gene with various honey bee and mouse tissues. All HVS mRNAs were expressed at various levels in the honey bee brain, thorax, and abdomen (Fig 1, Additional files 1 and 2). Ubiquitous expression was observed in the cases of *GB16549*, *GB13403*, *GB18344*, *GB18937*, and *GB16350 *(Fig. 1 and Additional file 2). Expressions of *GB16203*, *GB14232*, *GB15274*, and *GB17445 *mRNA were at least 2.5 times higher in the brain and abdomen than that in the thorax (Fig. 1). *GB15864 *and *GB17738 *mRNAs expression was 3 times higher in the brain and thorax than that in the abdomen, and the expression of *GB15370 *mRNA was weak in the abdomen (1.5 times less than that in the thorax) (Fig. 1). The remaining 60% of the honey bee HVS genes (18 out of 30) were expressed at a higher level in the brain than in the abdomen and thorax. In particular, the expressions of *GB14717*, *GB16908*, and *GB10273 *were exclusively detected in the brain (Additional file 2). *GB13871*, *GB19146*, *GB15533*, *GB19733*, and *GB18346 *mRNA expressions were 3.5, 5, 3, 2, and 3 times higher in the brain than in the abdomen, respectively (Fig. 1 and Additional file 2). Expressions of *GB11010*, *GB14271*, *GB18120*, *GB14468*, *GB18761*, *GB15999*, *GB17835*, *GB17260*, *GB18050*, and *GB10755 *mRNA were 3.5, 4, 4.5, 2.5, 7, 2, 1.5, 2, 1.5, and 2 times higher in the brain than in the thorax, respectively (Additional file 2).

**Figure 2:**
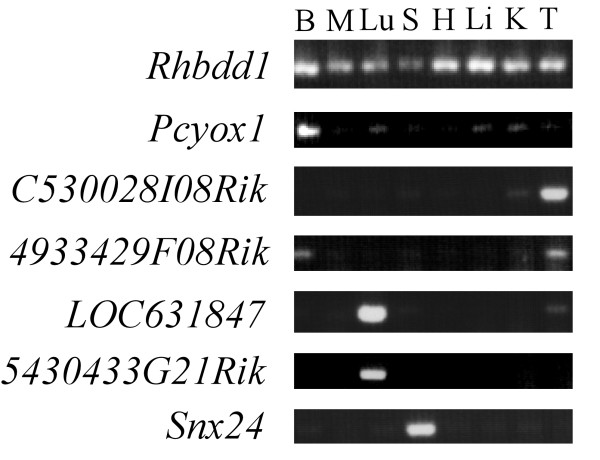
**Representative expression profiles of HVS genes in mouse tissues**. HVS mRNA expression levels in the mouse brain (B), muscle (M), lung (Lu), spleen (S), heart (H), liver (Li), kidney (K), and testis (T) were analyzed by semiquantitative RT-PCR. *Rhbdd1 *and other (total 11 genes) mRNAs are ubiquitously present in the above tissues. Expressions of *Pcyoxl *and *Pnrc1 *(Additional file 3) mRNAs and those of *C530028I08Rik *and *Rad18 *(Additional file 3) were abundant in the brain and testis, respectively. *4933429F08Rik *and other (total 7 genes) mRNAs were predominantly expressed in the brain and testis. See also Additional file 3. *LOC631847 *and *5430433G21Rik *mRNAs were primarily detected in the lung. *Snx24 *mRNA is expressed in the spleen. The remaining 20 mouse HVS genes show various "tissue-selective" expression patterns, as shown in Additional file 4.

The expression of mouse HVS genes in various tissues was also analyzed. For mouse HVS genes with more than 3 copies in the genome, the expression of 2 genes sharing the highest similarity with the honey bee gene was analyzed. Thus, we performed semiquantitative RT-PCR analysis with 45 mouse HVS genes (see Table 1). Most of the HVS genes showed tissue-selective expression patterns in mouse (Fig. 2, Additional file 3, and Additional file 4). Ubiquitous expression was observed with *Rhbdd1*, *Dnmt3a*, *Mtfr1*, *Armc1*, *Pnrc2*, *1300010M03Rik*, *Fbxlike3*, *Gemin7*, *Siva1*, *LOC434405*, and *Fbxo22 *(11 out of 45) (Fig. 2 and Additional file 3). The expressions of *Pcyoxl *and *Pnrc1 *were primarily detected in the brain (Fig. 2 and Additional file 3). *C530028I08Rik *and *Rad18 *were primarily expressed in the testis (Fig. 2 and Additional file 3). The expression levels of *4933429F08Rik*, *Dnmt3b*, *Cdca7*, *LOC217738*, *1700012P22Rik*, *2610016C23Rik*, and *Armc9 *mRNAs were high in both the brain and testis (Fig. 2 and Additional file 3). Thus, mouse HVS genes expressed in either or both the brain and testis accounted for a total of 11 (out of 45) genes. *LOC631847 *and *5430433G21Rik *mRNAs were highly expressed in the lung (Fig. 2). The expression of *Snx24 *was primarily detected in the spleen (Fig. 2). *Cdca7like *and *2010001M09Rik *mRNAs were expressed in the brain, lung, spleen, kidney, and testis but to a lesser extent in the muscle, heart, and liver (Additional file 4). *Snapc5 *and *Bcl2like1 *were expressed in all tissues examined, except in the muscle (Additional file 4). All other genes (16 out of 45) showed different tissue-selective expression patterns (Additional file 4).

### Expression profiles of HVS genes in mouse brain

**Figure 3:**
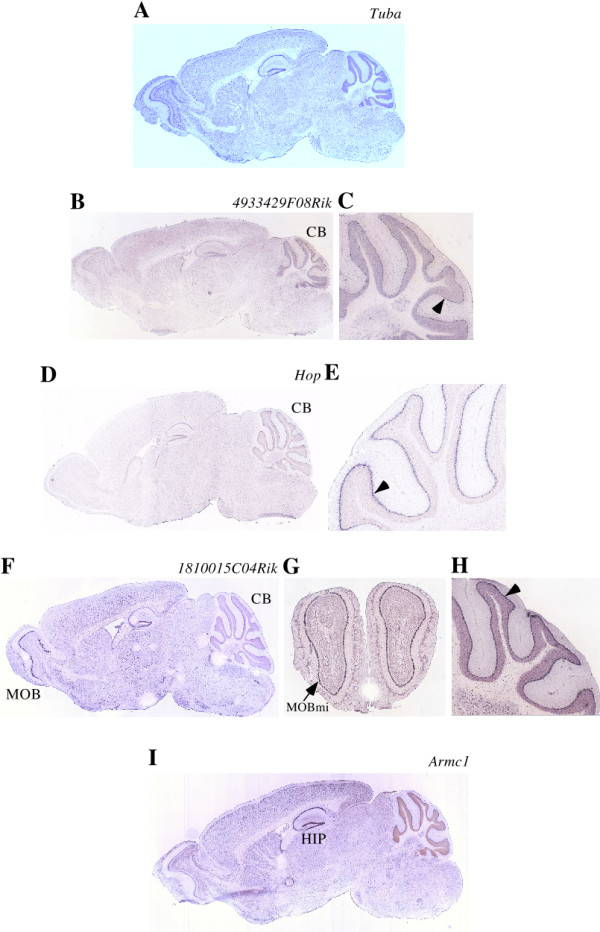
**Spatial expression patterns of *Tuba*, *4933429F08Rik*, *Hop*, *1810015C04Rik*, and *Armc1 *mRNAs in the adult mouse brain**. *Tuba *mRNA is relatively well expressed in the anterior part of the brain (A). Weak expression of *4933429F08Rik *mRNA is observed in the cerebellum (CB) (B), particularly in Purkinje cells (arrow head) (C). *Hop *mRNA is primarily detected in Purkinje cells (arrow head in E) of the cerebellum (CB) (D). Expression of *1810015C04Rik *mRNA is primarily detected in the olfactory bulb (MOB) and cerebellum (CB) (F). It is abundant in the mitral layer of the olfactory bulb (MOBmi in G) and in cerebellar Purkinje cells (arrow head in H). *Armc1 *mRNA is expressed throughout the brain; however, it is relatively well expressed in the hippocampus (HIP) (I).

Next, we characterized the spatial expression patterns of HVS genes in the mouse brain by searching data in the Allen Brain Atlas [28]. The atlas provides 2- and 3-dimensional images of the spatial expression patterns of ~20,000 genes in the adult mouse brain. *In situ *hybridization data are available for 34 mouse HVS genes. Among them, 20 genes exhibited only weak expression and, therefore, their expression could not be attributed to the specific cell types or brain regions. Neuron-enriched expression was detected for *Tuba*, *4933429F08Rik*, *Bcl2like1*, *Pcyox1*, *Pcyox1like*, *Hop*, *Armc1*, *Armc9*, *Pnrc1*, *1300010M03Rik*, *1810015C04Rik*, *Snapc5*, and *Rad18 *(Fig. 3 and Additional file 5). Only *Fbxo22 *was expression in both neurons and glial cells (Additional file 5A).

*Tuba *mRNA was expressed relatively well in the anterior part of the brain (Fig. 3A); its duplicated gene *4933429F08Rik *was, however, expressed in the cerebellum, particularly in Purkinje cells (Fig. 3B and 3C). The expression of *Hop *was primarily detected in cerebellar Purkinje cells (Fig. 3D and 3E). Expression of *1810015C04Rik *mRNA was abundant in the mitral layer of the olfactory bulb and in cerebellar Purkinje cells (Fig. 3F~H). The expression of *Armc1 *was ubiquitous throughout the entire brain; however, it was relatively well expressed in the hippocampus (Fig. 3I). *Pcyox1*, *Bcl2like1*, and *Pnrc1 *mRNAs were ubiquitously expressed in the brain, except for an increased hippocampal expression (Additional file 5B~D). *Pcyox1like*, *Armc9*, *1300010M03Rik*, *Rad18*, and *Snapc5 *mRNAs were uniformly expressed throughout the brain (Additional file 5E~I).

### Identification of genes specifically conserved between vertebrates and red flour beetle but lost from the other sequenced ecdysozoan genomes

**Figure 4:**
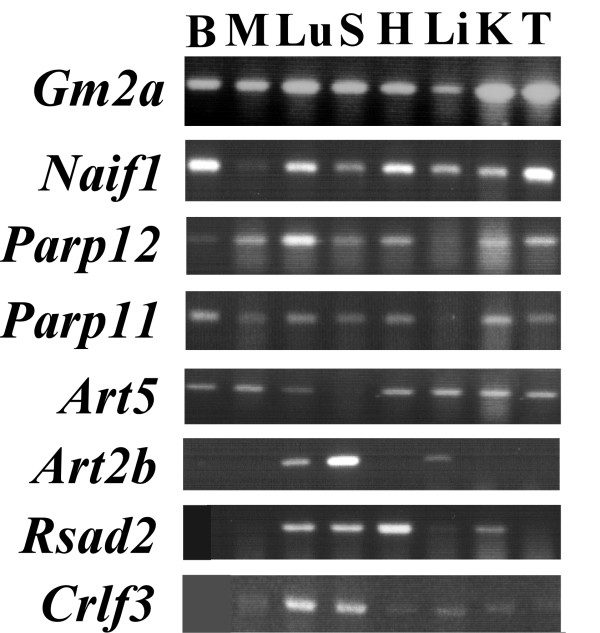
**Expression profiles of TVS genes in mouse tissues**. TVS mRNA levels in the mouse brain (B), muscle (M), lung (Lu), spleen (S), heart (H), liver (Li), kidney (K), and testis (T) were analyzed by semiquantitative RT-PCR, as shown in Fig. 2. Nearly ubiquitous expression was detected for the genes *Gm2a*, *Naif1*, *Parp12*, *Parp11*, and *Art5*. *Art2b *mRNA is expressed in the lung, spleen, and liver at high levels. *Rsad2 *mRNA is abundantly expressed in the lung, spleen, heart, and kidney. High levels of *Crlf3 *mRNA are expressed in the lung and spleen.

As controls, we used Urbilaterian genes that were uniquely shared between *T. castaneum *and vertebrate (TVS genes) identified in the same method as that used for HVS genes. In contrast to the 30 honey bee HVS genes, red flour beetle TVS genes were only 6 (Table 3). We also analyzed the expression patterns of 8 mouse TVS genes in various tissues as described above (Fig. 4). Nearly ubiquitous expression was observed with *Gm2a*, *Naif1*, *Parp12*, *Parp11*, and *Art5 *genes. *Art2b *was predominantly expressed in the lung, spleen, and liver; *Rsad2*, in the lung, spleen, heart, and kidney; and *Crlf3*, in the lung and spleen. Thus, none of the mouse TVS genes were expressed in either or both the brain and testis, unlike mouse HVS genes. Moreover, only 1 TVS gene, the poly (ADP-ribose) polymerase family was suggested to have been associated with gene expression in GO terms. Since only 8 mouse TVS genes were identified, it was not possible to statistically compare them with 45 mouse HVS genes. Nevertheless, these results support that honey bees retain more ancient Urbilaterian genes than red flour beetle.

**Table 3:** List of red flour beetle and mouse TVS genes

*Tribolium *GLEAN Model	Mouse Accession Number	E-value	Name/Comments	
Proteins with known domains				

GLEAN_08068	NP_034429	6.00E-16	Gm2a	GM2 ganglioside activator protein
GLEAN_11614	NP_067359	8.00E-121	Rsad2	radical S-adenosyl methionine domain containing 2
GLEAN_00209	NP_061246	3.00E-46	Crlf3	cytokine receptor-like factor 3
GLEAN_10124	NP_766481	1.00E-28	Parp12	poly (ADP-ribose) polymerase family, member 12
	NP_852067	3.00E-25	Parp11	poly (ADP-ribose) polymerase family, member 11
GLEAN_04003	NP_031517	2.00E-09	Art5	ADP-ribosyltransferase 5 precursor
	NP_064299	4.00E-07	Art2b	ADP-ribosyltransferase 2b

Proteins without known domains				

GLEAN_10286	NP_919316	1.00E-07	Naif1	nuclear apoptosis inducing factor 1

The sequences of *Tribolium *GLEAN models are available in BCM database .

## Discussion

### Evolution of HVS genes

Searching for HVS genes uniquely shared between vertebrates and honey bee resulted in the identification of 30 honey bee and 55 mouse genes. There are more HVS genes in mice than honey bees since multiple-copy HVS genes are often present in the mouse genomes as against single copy genes in the honey bee genome (Table 2). This finding suggested that Urbilateria had a relatively complex genome and that HVS genes have been lost from various ecdysozoans, which were shown to have more derived genomes than lophotrochozoan and deuterostome [1-7]. The loss and retention of these ancient genes in specific lineages has been implicated in generating metazoan diversity [3,4]; however, the causal relationships involved therein have not been extensively studied. There are 2 possibilities explaining the lineage-specific retention and loss of Urbilaterian genes. One possibility is that some genes are retained in specific lineages because during evolution, they may have had vital roles in these lineages but not in others. The other possibility is that many lineages evolved, for example, distant paralogs or gene network interactions, to compensate for the functions of Urbilaterian genes retained in the specific lineages, and the lineages eventually lost these genes. If the latter case is considered, some Urbilaterian genes must have been stochastically retained in specific lineages. We, thus, characterized HVS genes as a model to gain insights into the evolutionary mechanisms resulting in the presence of Urbilaterian genes only in specific lineages.

During the course of the identification of honey bee HVS genes, we first identified 40 genes shared between vertebrates and honey bee but not other sequenced insect (fly, mosquitoes, beetle, and moth) genomes. Furthermore, the nematode genome also lost 30 but retained 10 genes, demonstrating that its evolutionary rate was comparable to that of fruit fly and mosquito, as previously reported [29]. Ten genes shared between honey bee and nematode included a Malonyl-CoA decarboxylase responsible for the conversion of Malonyl-CoA to Acetyl-CoA, which is used in fatty acid biosynthesis as well as energy production. These functions should be very critical since this gene is conserved in bacteria and plants. The apparent lack of this enzyme in 5 insect species suggests that these species must have developed an alternative pathway for fatty acid metabolism. The functions of other genes shared between honey bee and nematode have not been reported.

Both sea urchin and ascidian contain a comparable number of HVS genes (Table 2); however, amino acid substitutions are more extensive in ascidian than in sea urchin. When the ascidian genome was searched for HVS genes by TBLASTN analysis using honey bee HVS genes as queries, only 16 genes with significant similarity were recovered. Consistent with previous reports [30,31], this result demonstrates that although ascidian is more closely related to the vertebrate lineage, it has a more derived genome than sea urchin. Honey bees contain a single copy for each HVS gene; however, vertebrates have multiple copies for 15 out of 30 (50%) honey bee HVS genes. This ratio is lower than the average percentage (approximately 70%, [32]) of genes having multiple copies in human and mouse. This may suggest that the loss of gene copy occurred more frequently with HVS genes after whole- or local-genome duplication events in vertebrates [33] (Table 2). The tissue expression patterns of HVS gene pairs are different in many cases (Fig. 2, Additional file 3, and Additional file 4), demonstrating that they either adopted a new function (neofunctionalization) or partitioned old functions (subfunctionalization). Among HVS genes, the copy-number expansions of genes encoding Bcl2 and TNF receptor superfamilies were detected in vertebrate genomes. Both Bcl2 and TNF receptor superfamilies control cell death and survival [24,34], a more extensive feature in vertebrates than in honey bee, sea urchin, and ascidian. Interestingly, among the vertebrates examined, the honey bee GB14271 orthologs were only present in mouse and human (mammals). This result suggests that gene loss also occurs in vertebrates, as reported previously [35,36].

To find the paralogs of HVS genes in the sequenced insect genomes, we first identified functional domains in each HVS protein by InterProScan  and collected these functional domain sequences from Ensembl. We, then, constructed the HMM models by HMMER 2 programs. Insect protein sets of Ensembl, Baylor, and the *Aedes *genome sequencing consortium were finally searched for the paralogs of HVS proteins by these HMMs with an E-value cutoff level of 1.0. An example indicates that the functions of HVS genes were compensated by the paralogs and/or gene network interactions. Both honey bees and vertebrate express *Dnmt1*, *2*, and *3*; however, members of the genus *Drosophila *express only *Dnmt2*, while those of *Tribolium *express *Dnmt1 *and *2*. Nonexpression of *Dnmt1 *and *3 *is consistent with the lack of CpG methylated DNA in *Drosophila *[37], and *Dnmt3 *expression is associated with the presence of CpG methylated DNA in honey bee [18]. It is well established that Dnmt3 is *de novo *DNA methyltransferase, and Dnmt1 is necessary for maintaining DNA methylation in vertebrates [25]. If CpG methylated DNA is present in the *Tribolium *genome, it can be inferred that Dnmt1, the paralog of Dnmt3, might also carry out *de novo *DNA methylation. Meanwhile, the epigenetic repression of gene transcription is functional in *Drosophila *through histone modifications, suggesting that *Drosophila *evolves the epigenetic control system independent of the methylated DNA by mechanisms such as modification of gene network interactions.

### Present functions of HVS genes appear to be biased towards expression in the brain and gonads

Most honey bee HVS genes (83%, 25 out of 30) were expressed at different levels in the brain, thorax, and abdomen (Fig. 1 and Additional file 2). Among them, 72% (18 out of 25) show a higher level of expression in the brain than in the thorax and abdomen, suggesting that their mRNAs appear to be enriched in the neurons. Most mouse HVS genes (76%, 34 out of 45) show tissue-specific or selective expression patterns (Fig. 2, Additional file 3, and Additional file 4). Among them, 32% (11 out of 34) are highly expressed in either or both the brain and testis. Intriguingly, 7 genes are highly expressed in both brain and testis. HVS genes are not essential for the survival of all animals since they are absent in many insect species other than honey bee. These genes are unlikely to have major roles for cell viability and maintenance and, thus, they were easily co-opted or recruited to function in the specific tissues, which in this case were mouse brain and testis. Both tissues contain a significant population of stem cells and, thus, the 7 HVS genes may have specific roles in regulating the proliferation and/or differentiation of these stem cells. Many genes associated with neuronal excitation and transmission are highly expressed in the brain but not in the testis. Meanwhile, genes involved in spermatogenesis are expressed exclusively in the testis and not in the brain. Genes highly expressed in both tissues are, therefore, not very common in mouse. It was reported that the total number of genes predominantly expressed in the human brain and testis is 664 out of the 7070 examined, accounting for only 9.4% [38]. Thus, the ancient Urbilaterian genes selectively retained between vertebrates and honey bee appear to be biased toward playing major roles in brain and gonad functions in mouse. In agreement with this finding, only 6 red flour beetle TVS genes (genes uniquely shared between red flour beetle and vertebrates) were identified, and no mouse TVS gene exhibited predominant expression either in the brain or testis (Table 3 and Fig. 4). We, therefore, would like to propose that at least some HVS genes might have been selectively maintained in order to support honey bee-specific brain and, possibly, gonad functions. It is very likely that brain functions involved in supporting social behavior and cognitive ability of honey bees and vertebrates evolve independently, without the sharing of any genetic components, on account of their evolutionary distance and the difference in their brain structures; however, selective genes of Urbilateria could be a part of the genetic bases underlying the evolution of advanced brain functions. We found that 13 mouse HVS genes were highly expressed in the neurons of the brain. Among them, 8 genes exhibited brain-region selective expression patterns. Most of them were highly expressed in the cerebellar and in the forebrain area, including the olfactory bulb, cerebral cortex, and hippocampus. These brain regions are responsible for the integration and processing of various sensory information as well as various forms of learning and memory. The roles of many other HVS genes in brain functions in both honey bees and mice remain to be determined.

The number of honey bee HVS genes is only 30, and this is consistent with the fact that gene loss and acquisition events are relatively rare as compared to amino acid substitution, *cis*-regulatory mutation, DNA rearrangement, and duplication during evolution. A vast majority of honey bee genes are, indeed, conserved in all solitary ecdysozoan genomes [15], suggesting that advanced honey bee brain functions are primarily driven by increasing complexity in the network of gene interactions. Since 7 out of 20 HVS gene products were predicted, by using GO terms, to be associated with transcription and modification of DNA as well as RNA processing in the nucleus, they could participate in such processes by regulating DNA transcription, splicing, and non-coding RNA synthesis. These HVS genes would provide an important resource for future studies on gene function.

## Conclusion

Large-scale comparison of metazoan genomes has revealed that a significant fraction of genes of Urbilateria is lost in each animal lineage. This event could be one of the underlying mechanisms responsible for metazoan diversity. We have found that HVS genes are biased towards expression in the brains and gonads of honey bees and mice, and 35% of HVS genes are associated with DNA transcription and RNA processing by GO analysis. These results suggest that HVS genes include genes that are biased towards expression in the brain and gonad. It also demonstrates that at least some of Urbilaterian genes are selectively retained in the specific animal lineage to support the species-specific phenotypes.

## Methods

### Identification of HVS genes

Protein sets were retrieved from Ensembl for *Drosophila*, *Anopheles*, and all vertebrates. *Tribolium *and *Apis *proteins were retrieved from Baylor and *Aedes *proteins from the *Aedes *genome sequencing consortium. Assignment to orthologous groups was performed as described earlier [39]. In short, we retained the longest ORF per locus and performed all-against-all comparisons using the Smith-Waterman algorithm. After grouping paralogous proteins, triangles of reciprocal best hits (involving 3 different species) were joined to build orthologous groups. Starting with a stringent cutoff, the joining was repeated with relaxed stringency in each successive step. All proteins in a group were required to have hits overlapping by at least 20 residues to avoid "domain walking" [17].

The candidate genes uniquely shared between honey bees and at least 1 abovementioned vertebrate were further screened by TBLASTN analysis with the genome and gene model (if available) DNA sequences of *D. melanogaster*, *A. gambiae*, *A, aegypti*, *B. mori*, and *T. castaneum*. Genes demonstrating a similarity to the abovementioned insect DNA sequences with an E value < E-03 were discarded. The resulting 40 genes were then analyzed by TBLASTN with the genome and gene model DNA sequences of *C. elegans*, as described above. Ten genes exhibited significant similarity (E value < 5E-04) to *C. elegans *genes, and were, therefore, eliminated. These methods resulted in the identification of 30 honey bee genes shared between at least 1 vertebrate and honey bee, but not any of the sequenced ecdysozoan genomes. We used the same strategy to identify genes uniquely shared between red flour beetle and at least 1 vertebrate (TVS genes).

### Distribution of HVS genes in deuterostome

The presence of the above HVS genes in sequenced deuterostome genomes (*S. purpuratus*, *C. intestinalis*, *T. nigroviridis*, *X. tropicalis*, *G. gallus*, *M. musculus*, and *H. sapiens*) was analyzed by TBLASTN, as described above. Genes exhibiting significant similarity (E value < E-03) to the honey bee HVS genes were scored at first. When homologous genes could not be identified in a particular species, their genomes were further analyzed by TBLASTN using HVS genes of other species (sea urchin, ascidian, and vertebrate) as queries. Once the homologous genes were identified in a particular species, they were used as queries to further screen for multiple-copy genes. If multiple-copy genes were identified, their locations on the contigs, scaffolds, and/or chromosomes were examined to verify them.

### Analysis of HVS genes by GO terms

We predicted HVS gene functions by assigning Gene Ontology (GO) terms (Gene-Ontology database, ). We searched for GO terms of each HVS gene using the AmiGO search engine. Search was conducted without filtering so that all GO terms were equally considered.

### RT-PCR analysis with honey bee and mouse tissues

Total RNA was isolated from the brain, thorax, and abdomen collected from 20 honey bee workers using Trizol reagent (Invitrogen). Similarly, total RNA was isolated from mouse brain, muscle, lung, spleen, heart, liver, kidney, and testis. Then, 5 μg of total RNA was used for reverse transcription reaction with ReverTra Ace reverse transcriptase (TOYOBO). The RT products were then used for PCR with Go Taq DNA polymerase (Promega). The annealing temperature was 5°C higher than the T_m _of the primers used, and the extension time was 30 s. PCR was repeated for 25 cycles, wherein the linearity between the band intensity and cycling number was confirmed. However, the cycling number to obtain amplified products was 30 for *Snx24*, *9230110C19Rik*, and *Art2b *and 35 for *GB14271*, *Tnfrsf4*, and *Naif1*. The RT-PCR products were sequenced to verify their identities. For honey bee HVS genes, the agarose gels were photographed, and the images were processed with Photoshop. The relative intensities of all bands detected for each gene were then measured with NIH image software (Image J). The data are shown in Additional file 1. The primer sequences used for PCR are available on request.

### Allen Brain Atlas data processing

The expression profiles of HVS genes in the mouse brain were classified according to the *in situ *hybridization and "heat map" (representing signal intensity) data [28]. The expression level in glia was examined by the signal intensity in the white matters of the various brain regions, such as cerebellum.

## Authors' contributions

TM and TY performed RT-PCR and Allen Brain Atlas data processing, SW and EMZ carried out bioinformatic analysis, and TK designed the experiments and wrote the manuscript.

## Supplementary Material

Additional file 1 **The relative values of each band intensity shown in Figure 1 and Additional file 2.** The data show the results of quantification of honey bee HVS mRNAs in the brain, thorax, and abdomen. Click here for file

Additional file 2 **Levels of other HVS mRNAs (not shown in Figure 1) in the honey bee brain, thorax, and abdomen were analyzed by semiquantitative RT-PCR.*** GB13403*, *GB18344*, *GB18937*, and *GB16350 *mRNAs are ubiquitously expressed (Ubiquitous). *GB14717*, *GB16908*, *GB11010*, *GB14271*, *GB18120*, *GB19146*, *GB10273*, *GB14468*, *GB18761*, *GB15533*, *GB15999*, *GB17835*, *GB19733*, *GB17260*, *GB18050*, *GB10755*, *GB18346 *mRNAs are highly expressed in the brain (Brain). Click here for file

Additional file 3 **Levels of other HVS mRNAs (not shown in Figure 2) in the mouse brain (B), muscle (M), lung (Lu), spleen (S), heart (H), liver (Li), kidney (K), and testis (T) were analyzed by semiquantitative RT-PCR.*** Dnmt3a*, *Mtfr1*, *Armc1*, *Pnrc2*, *1300010M03Rik*, *Fbxlike3*, *Gemin7*, *Siva1*, *LOC434405*, and *Fbxo22 *mRNAs are ubiquitously present (Ubiquitous). *Pnrc1 *and *Rad18 *mRNAs are predominantly expressed in the brain (Brain) and testis (Testis), respectively. *Dnmt3b*, *Cdca7*, *LOC217738*, *1700012P22Rik*, *2610016C23Rik*, and *Armc9 *mRNAs are present in the brain and testis in high levels (Brain and Testis). Click here for file

Additional file 4 **Levels of other HVS mRNAs (not shown in Figure 2 and Additional file 3) in the mouse brain (B), muscle (M), lung (Lu), spleen (S), heart (H), liver (Li), kidney (K), and testis (T) were analyzed by semiquantitative RT-PCR. ***Cdca7like *and *2010001M09Rik *mRNAs are primarily expressed in the brain, lung, spleen, kidney, and testis. *Snapc5 *and *Bcllike1 *are expressed in all tissues examined, except in the muscle. All other genes (16 out of 45), *Bcl2*, *Ccdc111*, *Snx22*, *Rtkn*, *Tuba*, *Pcyox1like*, *Hop*, *Bin3*, *Tnfrsf4*, *Tnfrsf14*, *Tmem45b*, *Tmem45a*, *Fbxlike21*, *1810015C04Rik*, *9230110C19Rik*, and *6430571L13Rik *show different tissue-selective expression patterns. Click here for file

Additional file 5 **Spatial expression patterns of *Fbxo22*, *Pcyox1*, *Bcl2like1*, *Pnrc1*, *Pcyox1like*, *Armc9*, *1300010M03Rik*, *Rad18*, and *Snapc5 *mRNAs in the adult mouse brain. ***Fbxo22 *mRNA is expressed in both neurons and glias throughout the brain (A). *Pcyox1 *(B), *Bcl2like1 *(C), *Pnrc1 *(D) mRNAs are ubiquitously expressed in the brain; however, they are abundantly expressed in the hippocampus (HIP). *Pcyox1like *(E), *Armc9 *(F), *1300010M03Rik *(G), *Rad18 *(H), and *Snapc5 *(I) mRNAs are ubiquitously present throughout the brain. Click here for file
