# Supplementary figures and images for: Expression profiles of urbilaterian genes uniquely shared between honey bee and vertebrates
Source: BMC Genomics. 2009 Jan 12;10:17. doi: 10.1186/1471-2164-10-17 (PMC2656531; doi:10.1186/1471-2164-10-17)

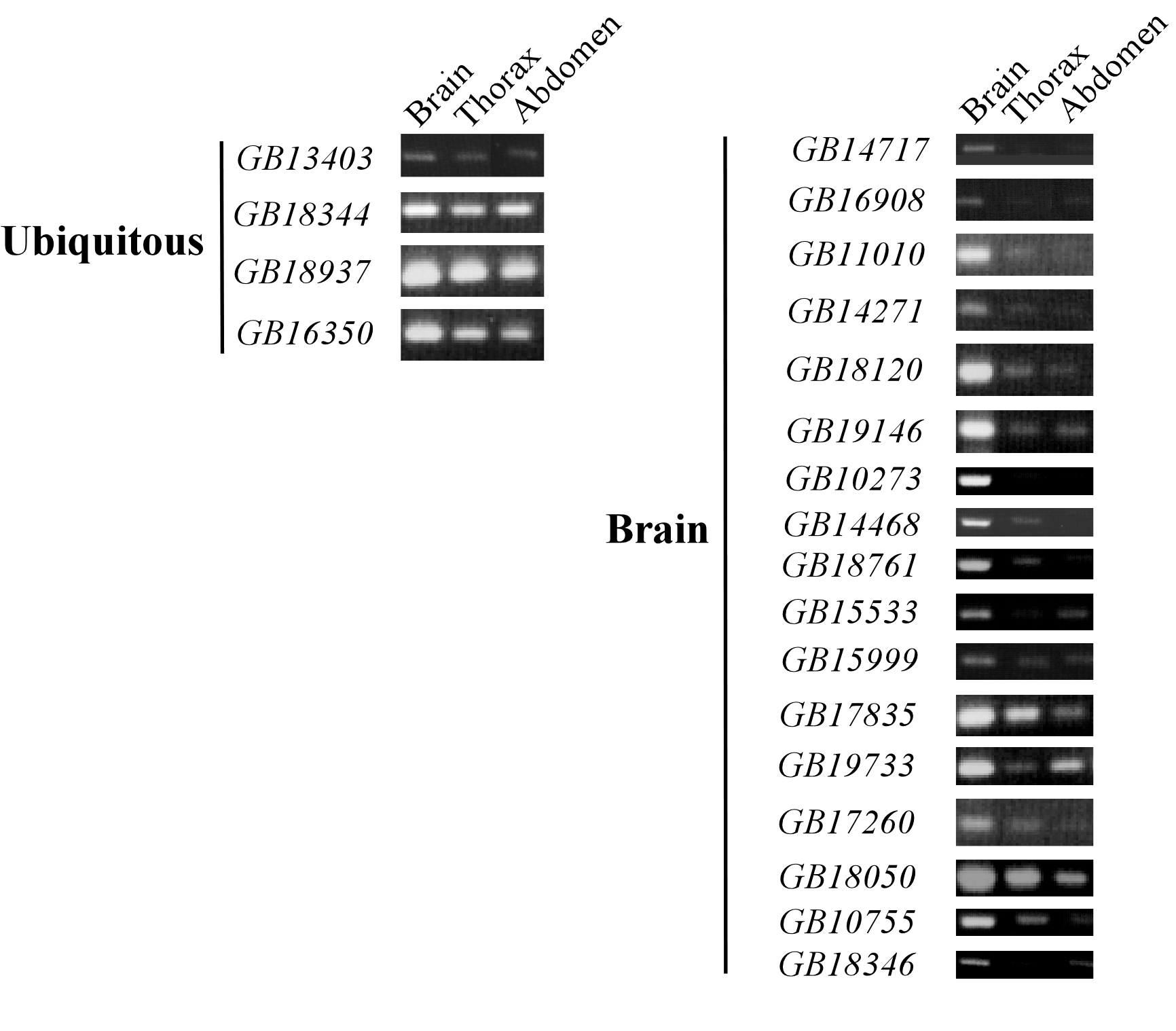

Supplement: Additional file 2 — Levels of other HVS mRNAs (not shown in Figure 1) in the honey bee brain, thorax, and abdomen were analyzed by semiquantitative RT-PCR. GB13403, GB18344, GB18937, and GB16350 mRNAs are ubiquitously expressed (Ubiquitous). GB14717, GB16908, GB11010, GB14271, GB18120, GB19146, GB10273, GB14468, GB18761, GB15533, GB15999, GB17835, GB19733, GB17260, GB18050, GB10755, GB18346 mRNAs are highly expressed in the brain (Brain). [file 1471-2164-10-17-S2.tiff]

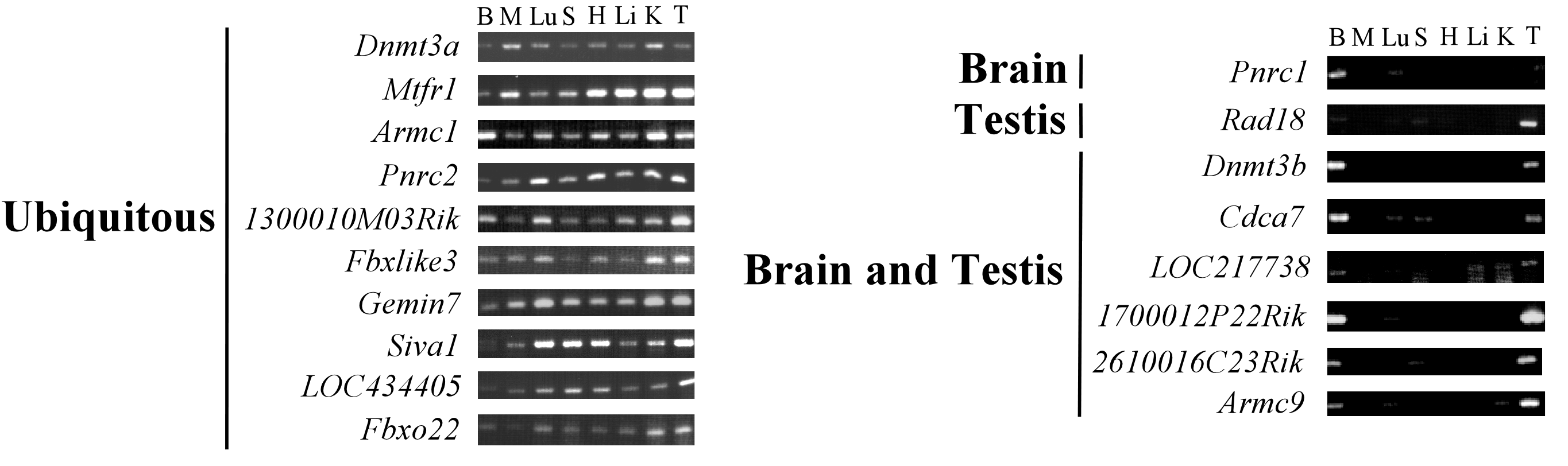

Supplement: Additional file 3 — Levels of other HVS mRNAs (not shown in Figure 2) in the mouse brain (B), muscle (M), lung (Lu), spleen (S), heart (H), liver (Li), kidney (K), and testis (T) were analyzed by semiquantitative RT-PCR. Dnmt3a, Mtfr1, Armc1, Pnrc2, 1300010M03Rik, Fbxlike3, Gemin7, Siva1, LOC434405, and Fbxo22 mRNAs are ubiquitously present (Ubiquitous). Pnrc1 and Rad18 mRNAs are predominantly expressed in the brain (Brain) and testis (Testis), respectively. Dnmt3b, Cdca7, LOC217738, 1700012P22Rik, 2610016C23Rik, and Armc9 mRNAs are present in the brain and testis in high levels (Brain and Testis). [file 1471-2164-10-17-S3.tiff]

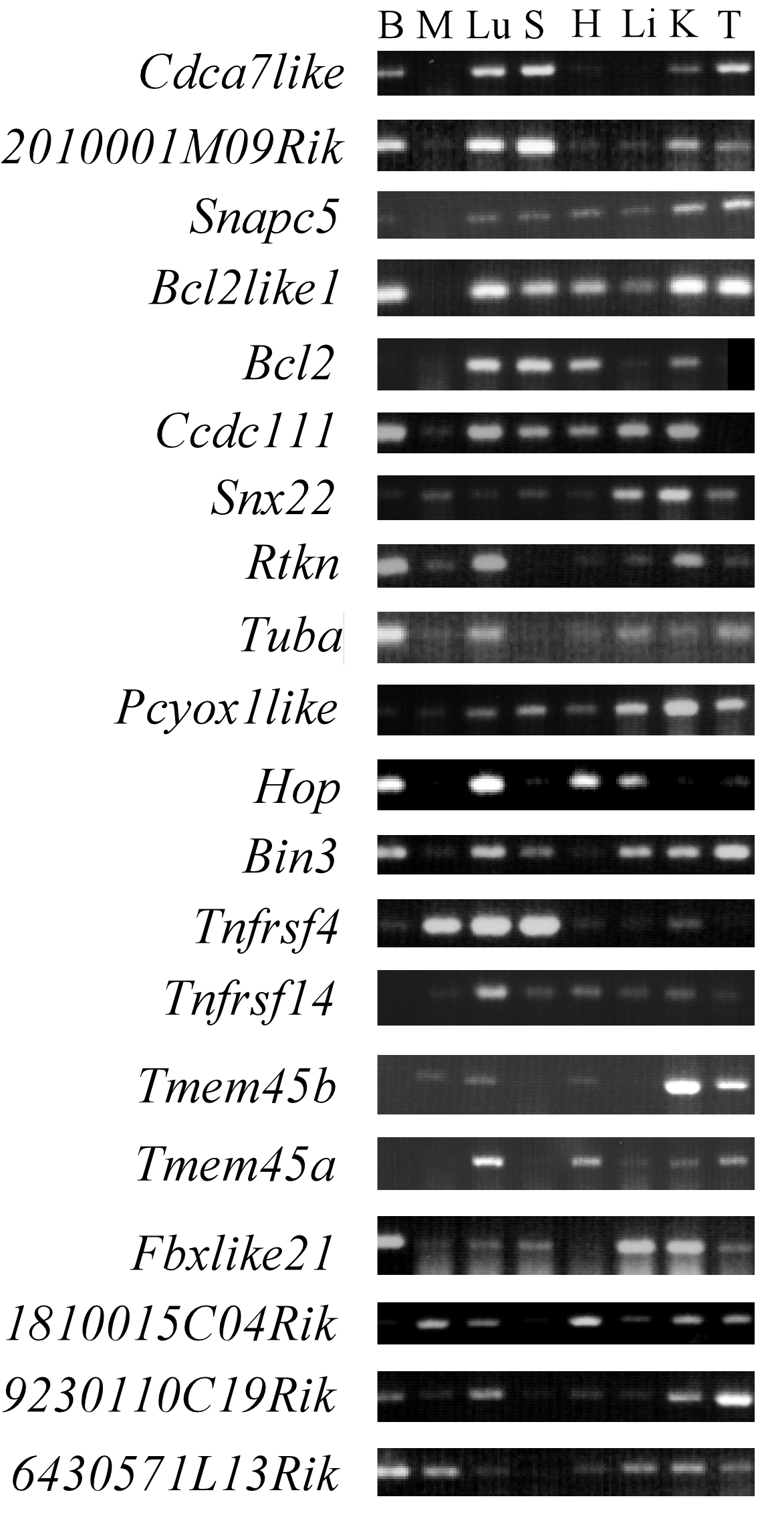

Supplement: Additional file 4 — Levels of other HVS mRNAs (not shown in Figure 2 and Additional file 3) in the mouse brain (B), muscle (M), lung (Lu), spleen (S), heart (H), liver (Li), kidney (K), and testis (T) were analyzed by semiquantitative RT-PCR. Cdca7like and 2010001M09Rik mRNAs are primarily expressed in the brain, lung, spleen, kidney, and testis. Snapc5 and Bcllike1 are expressed in all tissues examined, except in the muscle. All other genes (16 out of 45), Bcl2, Ccdc111, Snx22, Rtkn, Tuba, Pcyox1like, Hop, Bin3, Tnfrsf4, Tnfrsf14, Tmem45b, Tmem45a, Fbxlike21, 1810015C04Rik, 9230110C19Rik, and 6430571L13Rik show different tissue-selective expression patterns. [file 1471-2164-10-17-S4.tiff]

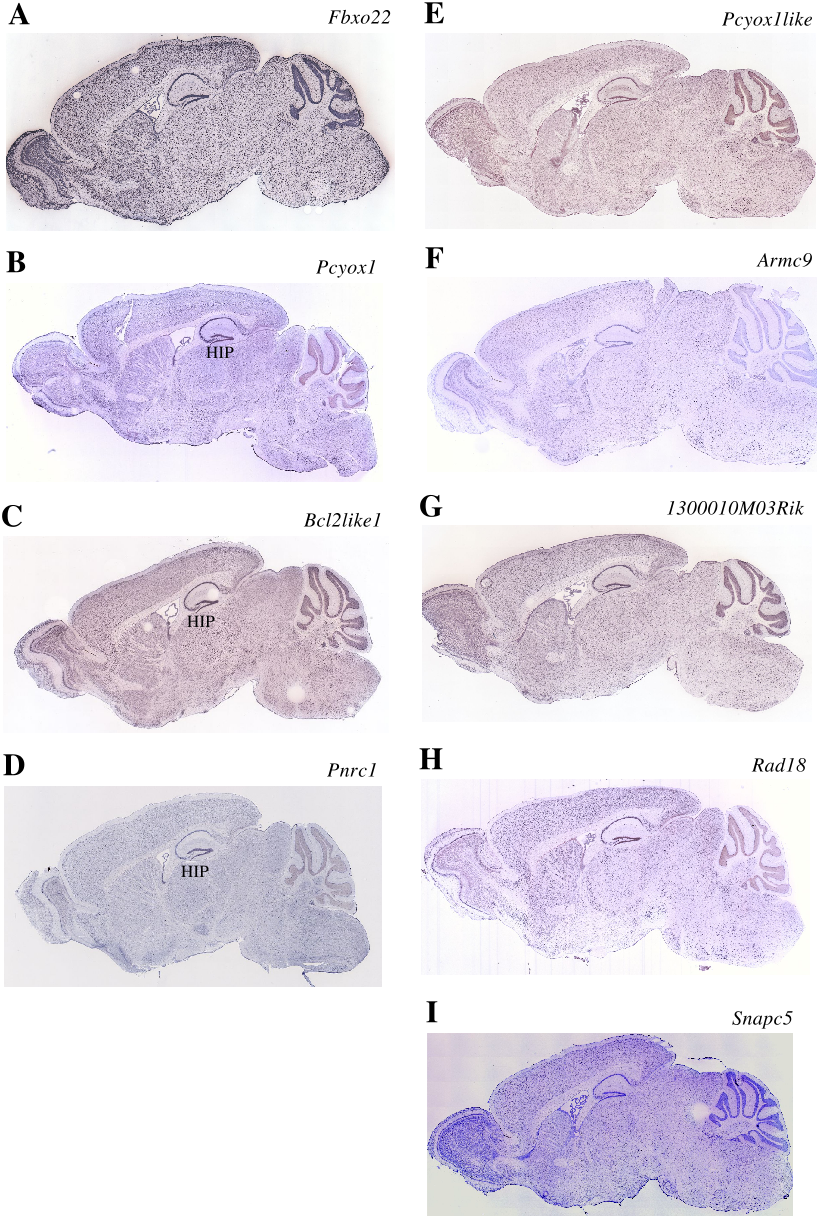

Supplement: Additional file 5 — Spatial expression patterns of Fbxo22, Pcyox1, Bcl2like1, Pnrc1, Pcyox1like, Armc9, 1300010M03Rik, Rad18, and Snapc5 mRNAs in the adult mouse brain. Fbxo22 mRNA is expressed in both neurons and glias throughout the brain (A). Pcyox1 (B), Bcl2like1 (C), Pnrc1 (D) mRNAs are ubiquitously expressed in the brain; however, they are abundantly expressed in the hippocampus (HIP). Pcyox1like (E), Armc9 (F), 1300010M03Rik (G), Rad18 (H), and Snapc5 (I) mRNAs are ubiquitously present throughout the brain. [file 1471-2164-10-17-S5.tiff]
